# Charakterisierung der Vorhofflimmerlast mittels Wearables

**DOI:** 10.1007/s00399-024-00995-2

**Published:** 2024-02-09

**Authors:** Konstanze Betz, Dominik Linz, David Duncker, Henrike A. K. Hillmann

**Affiliations:** 1 Department of Cardiology and Cardiovascular Research Institute Maastricht (CARIM), Maastricht University Medical Center, Universiteitssingel 50, 6229 ER Maastricht, Niederlande; 2 Klinik für Innere Medizin, Eifelklinik St. Brigida GmbH & Co. KG, Simmerath, Deutschland; 3 Netherlands Heart Institut, Utrecht, Niederlande; 4 Centre for Heart Rhythm Disorders, University of Adelaide and Royal Adelaide Hospital, Adelaide, Australien; 5 Department of Biomedical Science, Faculty of Health and Medical Sciences, University of Copenhagen, Kopenhagen, Dänemark; 6 Hannover Herzrhythmus Centrum, Klinik für Kardiologie und Angiologie, Medizinische Hochschule Hannover, Hannover, Deutschland

**Keywords:** Vorhofflimmern, Tragbare digitale Geräte, Photoplethysmographie, 1‑Kanal-EKG, Digitale Medizin, Atrial fibrillation, Wearable electronic devices, Photoplethysmography, 1‑channel ECG, Digital health

## Abstract

Die Charakterisierung von Vorhofflimmern (VHF) entsprechend der aktuellen Leitlinien bezieht sich kategorisch auf die Differenzierung zwischen paroxysmalem, persistierendem und permanentem VHF. Eine genauere Charakterisierung, auch mithilfe einer Evaluation der Vorhofflimmerlast, spielt sowohl in der Wissenschaft als auch im klinischen Alltag eine zunehmende Rolle. Wearables, insbesondere mit der Möglichkeit einer passiven (semi-)kontinuierlichen Aufzeichnung, können hier zur genaueren Quantifizierung beitragen. Primär bei Patient:innen mit bereits etablierter Vorhofflimmerdiagnose kann die Evaluation der Vorhofflimmerlast beispielsweise zur Erfolgskontrolle einer antiarrhythmischen Therapie, sei es medikamentös oder interventionell, eingesetzt werden. Offen bleiben jedoch noch wichtige Fragestellungen: Neben einer einheitlichen, evidenzbasierten Definition der Vorhofflimmerlast müssen auch klinisch relevante Cut-off-Werte sowie daraus resultierende therapeutische Konsequenzen (z. B. eines subklinischen Vorhofflimmerns) erarbeitet werden. Zudem sollte eine Etablierung und Evaluation von Versorgungsstrukturen zur Auswertung und klinischen Anwendung der Vorhofflimmerlast, insbesondere unter Einbezug von mittels Wearables erhobenen Daten, stattfinden.

## Abstract (en)

The characterization of atrial fibrillation (AF) according to current guidelines categorically refers to the differentiation between paroxysmal, persistent, and permanent AF. A more precise characterization of AF, including the evaluation of AF burden, is playing an increasingly significant role in both scientific research and clinical practice. Digital devices, especially those with the capability of passive (semi-)continuous recording, can contribute to a more accurate quantification of AF burden. Particularly in patients with an already established diagnosis of AF, the evaluation of AF burden can be used to monitor the success of antiarrhythmic therapy including antiarrhythmic drugs or pulmonary vein isolation. However, important questions remain unanswered: In addition to a uniform, evidence-based definition of AF burden, clinically relevant cut-offs for AF burden and resulting therapeutic consequences (e.g., subclinical AF) need to be elaborated. Furthermore, the establishment and evaluation of care structures for assessing and integrating AF burden in clinical care, especially by incorporating data from wearable medical devices, should take place.

Wearables, tragbare digitale Geräte, dienen der Überwachung des Herzrhythmus und der Erkennung von Herzrhythmusstörungen. Sie haben in den letzten Jahren an Bedeutung gewonnen und sind zum Zweck der Herzrhythmusüberwachung heute bereits gut im klinischen Alltag etabliert. Empfohlen werden sie von den Fachgesellschaften zum aktuellen Zeitpunkt insbesondere zum Vorhofflimmerscreening sowie zur Rhythmus-Symptom-Korrelation bei vermuteten Herzrhythmusstörungen [1, 2]. Die klinisch relevante Charakterisierung von VHF, nach der aktuell Therapieentscheidungen getroffen werden, bezieht sich kategorisch auf die Differenzierung zwischen paroxysmalem, persistierendem und permanentem VHF. Eine genauere Charakterisierung des VHF durch den dynamischen, progressiven Charakter der Vorhoferkrankung und das neue pathophysiologische Konzept der atrialen Kardiomyopathie, rücken in der letzten Zeit vermehrt in den klinischen und wissenschaftlichen Fokus [3–5]. Neben verschiedenen elektrokardiographischen, bildgebenden und biomarkerbasierten Verfahren kann auch die Bestimmung der Vorhofflimmerlast eine weitere, im Vergleich zur kategorischen Einteilung genauere, Differenzierungsmöglichkeit des VHF bieten. Wearables können zur Charakterisierung der Vorhofflimmerlast herangezogen werden.

## Wearables zur Arrhythmiediagnostik – Grundlagen

**Abb. 1:**
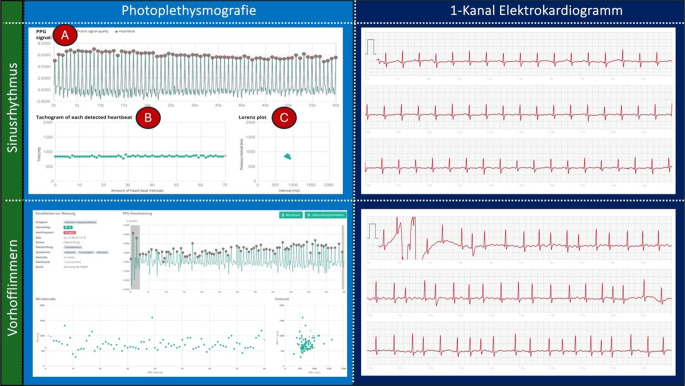
Wearable-basierte Technologien zur Herzrhythmusüberwachung. Vergleich zwischen der grafischen Darstellung einer Sinusrhythmus- und einer Vorhofflimmerepisode, aufgezeichnet mittels Photoplethysmographie und 1‑Kanal-EKG. Die Darstellung mittels Photoplethysmographie (PPG) setzt sich zusammen aus einem PPG-Signal (*A*), einem Tachogramm (*B*) und einem Poincaré bzw. Lorenzplot (*C*). Das PPG-Signal zeigt die Herzrhythmusaufzeichnung über 60 s. Einzelne Schläge sind *rot markiert* und zeigen die Frequenz pro Minute an. Der Zyklus wird durch die zeitliche Distanz zweier aufeinander folgender Schläge (*rote Punkte*) berechnet. Das Tachogramm zeigt die Zeitintervalle (*Y‑Achse*) der aufeinanderfolgenden Pulssignale (*X‑Achse*). Der Poincaré/Lorenzplot stellt die Pulszykluslänge zusätzlich als Funktion des vorangegangenen Intervalls dar [6]

Der Begriff Wearable umfasst unterschiedliche Gerätetypen. Hierzu zählen Smartphones mit zugehöriger Appfunktion, Smartwatches, Armbänder sowie andere tragbare Geräte, jeweils mit integrierten Sensoren, die der Herzrhythmusaufzeichnung und -interpretation dienen. Je nach Wearable werden unterschiedliche Technologien genutzt. Hier unterscheidet man bezüglich der Herzrhythmusüberwachung insbesondere das Elektrokardiogramm (EKG) von der Photoplethysmographie (PPG; Abb. 1).

Bei Wearables mit integrierter EKG-Funktion kann, je nach genutztem Wearable, ein 1‑Kanal- bis hin zu einem 12-Kanal-EKG aufgezeichnet werden. Aktuellen Leitlinien folgend, kann die Diagnose Vorhofflimmern (VHF) anhand eines 30-sekündigen 1‑Kanal-EKG gestellt werden [2]. Die PPG-Technologie basiert auf der Messung von Lichtabsorption. Ein im Wearable eingebauter Sensor misst die im Blut reflektierten Lichtimpulse einer auf die Haut gerichteten Lichtquelle und zeichnet diese grafisch als Kurve auf. Über die Variation der reflektierten Impulse, die der Pulsvariation entsprechen, können so Veränderungen im Herzrhythmus erkannt und der Verdacht auf die zugrunde liegende Herzrhythmusstörung gestellt werden [6, 7]. Laut aktuellen Leitlinien bedarf die Diagnosestellung von VHF und anderen Arrhythmien einer EKG-Dokumentation [8]. Daher muss jede PPG-dokumentierte Vorhofflimmerepisode bei einer Person, bei der bislang kein VHF diagnostiziert wurde, durch eine EKG-Dokumentation bestätigt werden. Jedoch kann durch eine große Anzahl an normalen PPG-Aufnahmen, aufgrund der hohen Sensitivität und Spezifität, [9, 10] ein VHF relativ sicher ausgeschlossen werden.

Weiterhin wird zwischen aktiver und passiver Überwachung unterschieden: Im Rahmen der aktiven Überwachung wird die Aufzeichnung durch die Patient:innen selbst ausgeführt, während bei der passiven Überwachung eine Aufzeichnung beim Tragen des Devices ohne (aktive) Unterstützung der Nutzer erfolgt. Eine aktive Überwachung ermöglicht punktuelle Aufzeichnungen, während eine passive Überwachung die Möglichkeit zur (semi-)kontinuierlichen Aufzeichnung bietet. Eine Übersicht häufig genutzter Wearables und deren Eigenschaften ist in Tab. 1 dargestellt.

**Tab. 1:** Übersicht und Eigenschaften im Klinikalltag häufig genutzter Wearables

Wearable	EKG (mögliche Ableitungen)	PPG	Aktiv	Passiv
Smartwatch	+ (1)	+	+	+
Smartphone	–	+	+	–
Smart Ring	–	+	–	+
Tragbares Daumen-EKG (Kardia)	+ (1–6)	–	+	–
Aufklebbare Patches	+	–	–	+^a^

*EKG* Elektrokardiogramm, *PPG* Photoplethysmographie

^a^Passive Aufzeichnung nur für eine begrenzte Dauer (i. d. R. 7–14 Tage) möglich

## Charakterisierung der Vorhofflimmerlast – Stellenwert im klinischen Alltag

Die Detektion von VHF kann EKG-basiert oder Device-basiert erfolgen: Durch implantierbare Schrittmacher mit einer Vorfhofsonde kann die Gesamtdauer in atrialen Hochfrequenzepisoden (AHRE) und durch implantierbare Loop-Rekorder kann die Zeit mit VHF während der Beobachtungszeit evaluiert werden (Device-detektiertes VHF; [2]). Die Interpretation von AHRE als Surrogatparameter für VHF sollte aufgrund einer hohen Rate an falsch-positiven Ereignissen [11] unter Vorsicht erfolgen und bedarf einer visuellen Beurteilung der Aufnahmen. Die klinische Vorhofflimmerlast wird hingegen mittels temporärer Überwachungsformen, wie intermittierender EKG-Überwachung (EKG-detektiertes VHF), charakterisiert. Eine langzeitliche, intermittierende Überwachung von VHF-Episoden mittels Wearables zeigte in Studien eine effektivere Detektion von VHF-Rezidiven im Vergleich zu kurzzeitigem, kontinuierlichem Holter-Monitoring [12]. Weiterhin ergab ein Vergleich verschiedener Screening-Strategien zur Erkennung von VHF bei Personen mit Schlaganfallrisikofaktoren, dass der diagnostische Ertrag mit zunehmender Dauer, Verteilung und Anzahl der Screenings zunahm, wobei alle VHF Screeningstrategien einen, im Vergleich zum implantierten Loop-Rekorder, geringeren diagnostischen Ertrag aufwiesen [13].

Weitere Studien zur Vereinheitlichung vorhandener Definitionen sowie die Suche nach weiteren, möglicherweise geeigneteren Methoden zur Messung der Vorhofflimmerlast, sind zum aktuellen Zeitpunkt noch ausstehend [2, 14].

Der Umgang mit Device-detektiertem VHF bei Patient:innen mit bislang noch nicht EKG-diagnostiziertem klinischem VHF wird weiterhin aktiv diskutiert. Neben Post-hoc-Analysen [15–17], die bei Patient:innen mit persistierendem im Vergleich zu paroxysmalen VHF ein erhöhtes Schlaganfallrisiko aufzeigen, weisen auch andere Studien auf ein erhöhtes Risiko bei Patient:innen mit AHRE hin [18–20]. Die kürzlich publizierten Ergebnisse der NOAH-AFNET-6-Studie [21] zeigten bei Patient:innen ohne bekanntes VHF mit dem Zufallsbefund von AHRE keine signifikante Senkung des kumulativen Endpunkts bestehend aus Schlaganfall, systemischen Embolien oder kardiovaskulärem Tod durch orale Antikoagulation (OAK) mittels Edoxaban im Vergleich zu Placebo, unabhängig von der Episodendauer [21, 22]. Es zeigte sich ein erhöhtes Risiko für schwere Blutungsereignisse [21]. Daten der randomisierten ARTESiA-Studie ergaben bei Patient:innen mit subklinischem VHF und OAK (Apixaban) im Vergleich zu einer Kontrollgruppe mit Aspirin, ein geringeres Risiko für Schlaganfall und systemische Thromboembolien, bei jedoch erhöhtem Risiko für schwere Blutungsereignisse [23]. Eine Metaanalyse der beiden randomisierten Studien (NOAH-AFNET 6 und ARTESiA) mit insgesamt 6548 Patient:innen zeigte eine Konsistenz der Studienergebnisse mit hoher Qualität der Evidenz: Eine orale Antikoagulation (Edoxaban oder Apixaban) reduzierte das Risiko für ischämischen Schlaganfall bei Patient:innen mit Device-detektiertem VHF signifikant, jedoch einhergehend mit einem erhöhten Risiko für schwere Blutungen [24].

Aktuelle Leitlinien empfehlen zur Indikationsstellung einer Antikoagulation bei Patient:innen mit VHF die Evaluation des vaskulären Risikoprofils anstelle der Vorhofflimmerlast [2, 14]. Weiterhin haben bisherige Interventionen zur Reduktion der Vorhofflimmerlast auch hinsichtlich einer Verbesserung der Lebensqualität keinen Erfolg zeigen können [25–27]. Eine höhere Vorhofflimmerlast ist jedoch mit einem höheren Sterblichkeitsrisiko assoziiert [28]. Erneut ist die Datenlage hier jedoch bezüglich AHRE ungenau.

Insbesondere in Bezug auf das erhöhte Mortalitätsrisiko sollte die Vorhofflimmerlast trotz der bleibenden Wissenslücken gesenkt werden. Analog zur Therapie des VHF ist eine strukturierte und umfassende Behandlung von atherosklerotischen Risikofaktoren, inklusive dauerhafte Reduktion von Übergewicht, dabei effektiv [29, 30]. Die Datenlage hinsichtlich der Effekte von körperlichem Training, intensiver Blutdrucksenkung und Stressreduktion auf Senkung der Vorhofflimmerlast ist derzeit noch nicht einheitlich [14].

Neben der (Erst‑)Detektion von Vorhofflimmern hat auch die Rezidivsuche nach erfolgter antiarrhythmischer Therapie einen relevanten Stellenwert im klinischen Alltag. Auch hier wird aktuell noch diskutiert, inwiefern eine genauere Charakterisierung der Vorhofflimmerlast im Vergleich zur kategorischen Identifizierung von Rezidiven therapeutische und/oder prognostische Vorteile bringen kann. Kontinuierliches Rhythmusmonitoring vor einer Ablation sagte in einer Subanalyse der CIRCA-DOSE-Studie die Vorhofflimmerlast genauer voraus und reflektierte die Freiheit von VHF-Rezidiven nach Ablation besser als die klinische Einschätzung [31]. Eine Sekundäranalyse basierend auf dem gleichen Studienprotokoll (CIRCA DOSE) ergab zudem, dass die klinische Klassifikation des VHF möglicherweise nicht ausreichend die Ergebnisse nach Ablation widerspiegelt, da Patient:innen mit VHF-Episoden von weniger als 24 h in einem kontinuierlichen Rhythmusmonitoring eine signifikant niedrigere Inzidenz von VHF-Rezidiven nach der Ablation aufwiesen [32].

Offen bleibt, ob die Vorhofflimmerlast als Marker der Schwere und des Progresses der atrialen Kardiomyopathie gesehen werden kann. Prospektive Daten aus größeren Kohorten können hier ausschlaggebend sein. Wearables können zudem eine Möglichkeit der (semi-)kontinuierlichen Erfassung der Vorhofflimmerlast bieten [14].

## Stellenwert von Wearables bei der Evaluation der Vorhofflimmerlast

Wie oben beschrieben, erfolgen Evaluationen der Vorhofflimmerlast aktuell hauptsächlich mit Hilfe von (semi-)kontinuierlichen EKG-Aufzeichnungen, beispielsweise mittels Langzeit-EKG oder im Rahmen von Kontrollen implantierter Schrittmacher- oder Defibrillatoren in Form von AHRE Device-basiert. Ferner können auch implantierbare Loop-Rekorder zum kontinuierlichen Rhythmusmonitoring, beispielsweise nach erfolgter Ablation, zur Evaluation der postprozeduralen Vorhofflimmerlast genutzt werden [2]. Neben den genannten Devices erscheint auch der Einsatz von Wearables zur Bestimmung der Vorhofflimmerlast sinnvoll.

**Abb. 2:**
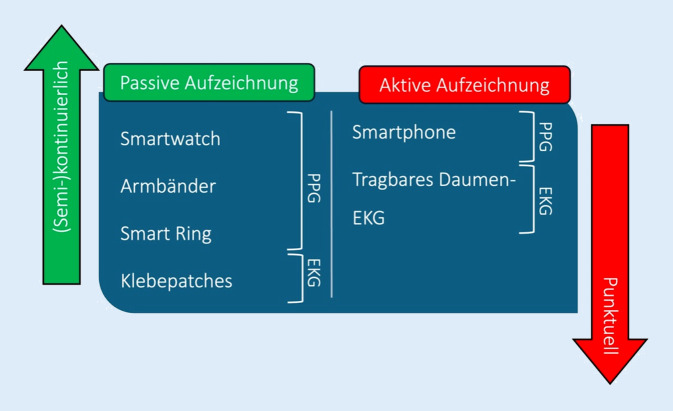
Potenzielle Wearables zur Charakterisierung der Vorhofflimmerlast. Während aktive Aufzeichnung aufgrund der punktuellen Dokumentation nur eine Momentaufnahme darstellen, ist es mittels passiver Aufzeichnung möglich, eine (semi-)kontinuierliche Aufzeichnung und somit eine Quantifizierung der Vorhofflimmerlast vorzunehmen

Aufgrund der punktuellen Rhythmusaufzeichnung und -überwachung sollten Wearables mit ausschließlich aktiver Überwachungsfunktion zur Charakterisierung der Vorhofflimmerlast nicht eingesetzt werden. Sinnvoll ist der Einsatz von Wearables mit passiver (semi-)kontinuierlicher Überwachungsfunktion (Abb. 2).

Im Gegensatz zu herkömmlichen Verfahren wie der Nutzung eines Langzeit-EKG besteht hier die Möglichkeit einer längerfristigen und dadurch ggf. genaueren Beurteilung der Vorhofflimmerlast. Voraussetzung ist eine engmaschige (semi-)kontinuierliche Aufzeichnung. Wearables mit passiver Aufzeichnungsmöglichkeit basieren häufig auf PPG-Messungen, da eine EKG-Messung bei den meisten Devices eine aktive Initiierung durch PatientInnen voraussetzt. In einer kürzlich publizierten Studie zur Machbarkeit und Genauigkeit eines PPG-Monitorings mittels eines Armbands identifizierte die PPG-basierte Methode die Vorhofflimmerlast im Vergleich zum Holter-Monitoring in 85,1 % der Fälle korrekt [33]. Auch das Smartwatch-basierte Monitoring zeigte eine hohe Sensitivität für die Detektion von Vorhofflimmerauftreten sowie die Vorhofflimmerdauer im Vergleich zu implantierbaren Rekordern [34]. Dennoch zeigen Studien auch mögliche Probleme der PPG-basierten Erfassung der Vorhofflimmerlast auf. Eine absolute Quantifizierung kann durch artefaktüberlagerte Signale erschwert werden, was insbesondere in der breiten alltäglichen Anwendung der Wearables ein Problem darstellen kann [33]. In verschiedenen Analysen zeigten sich so etwa 50 % der PPG-Daten auswertbar [33–35]. Weiterhin können atriale/ventrikuläre Extrasystolen zu falsch-positiven, Vorhofflattern mit regelmäßiger Frequenz zu falsch-negativen Ergebnissen führen [33]. Eine Anpassung des auswertenden Algorithmus hin zu Robustheit gegenüber verrauschten Signalen kann die Sensitivität von PPG-basierten Wearables verbessern [36]. Weiterhin ist eine individuelle Anpassung der Wearables nach Größe und Material eine Möglichkeit, um die Aufnahmequalität zu steigern [33].

Laut aktuellen Empfehlungen ist bei mittels PPG gestelltem Verdacht auf VHF zwar eine EKG-Dokumentation vor Diagnosestellung notwendig – bei bereits etablierter Vorhofflimmerdiagnose erscheint eine Quantifizierung der Vorhofflimmerlast mittels PPG-Technologie sinnvoll, wenn dies auch in den aktuellen Empfehlungen bisher aufgrund der geringen Datenlage nicht abgebildet ist. Mögliche Anwendungsbereiche für die Bestimmung der Vorhofflimmerlast über Wearables können die postprozedurale Überwachung nach Vorhofflimmerablation zur Bestimmung asymptomatischer Rezidive sein [2, 37, 38]. Eine aktuelle Studie zeigte hier eine Assoziation zwischen mittels PPG detektierter Vorhofflimmerepisoden innerhalb der ersten Woche nach Pulmonalvenenisolation und Langzeitrezidiven [39]. Auch möglich ist die Evaluation einer Symptom-Rhythmus-Korrelation zur Differenzierung der Symptomatik basierend auf Vorhofflimmerepisoden oder z. B. anderer Arrhythmien ohne Ablationsperspektive [2]. Weiterhin bietet das Monitoring der Vorhofflimmerlast mittels Wearables auch Möglichkeiten zur außerklinischen Überwachung einer rhythmus- bzw. frequenzkontrollierenden Therapie. Aus wissenschaftlicher Perspektive bringt die potenzielle Zunahme an (semi-)kontinuierlicher Rhythmusüberwachung die Möglichkeit, die Vorhofflimmerlast in breiten Bevölkerungsgruppen (z. B. im niedrigen Risikobereich) sowie über einen längeren zeitlichen Verlauf und hinsichtlich klinischer Implikationen, wie z. B. dem Umgang mit der Detektion von subklinischem VHF, besser analysieren und verstehen zu können [14]. Es bleiben jedoch einige grundsätzliche Fragen offen, die vor einer breiten Anwendung evaluiert werden müssen.

## Offene Fragen

Die drängendsten Fragen zeigen sich in Hinblick auf die Evidenz und Evaluation einer einheitlichen Definition der Vorhofflimmerlast sowie bezüglich der richtigen Messmethodik. Darauf aufbauend sollten Cut-off-Werte für eine klinisch relevante Vorhofflimmerlast definiert und evaluiert werden [14]. Letzteres baut jedoch auf einem besseren Verständnis der zugrundeliegenden pathophysiologischen Mechanismen hinter der Vorhofflimmerlast auf. Dies kann zu einer besseren Interpretation führen und somit womöglich auch offene klinische Fragen, beispielsweise nach kardialen oder vaskulären Risiken bei einer erhöhten Vorhofflimmerlast, beantworten. Wearables bieten die Möglichkeit, durch (semi-)kontinuierliche Aufzeichnungen in breiten Bevölkerungsgruppen wichtige Informationen zu generieren und prospektiv zu untersuchen [14]. Eine breite Verfügbarkeit und Generierung von (semi-)kontinuierlichen Rhythmusdaten kann jedoch zu neuen Problemen in den Versorgungsstrukturen führen, über die rechtzeitiger Konsens geschaffen werden muss. So sollte der Umgang mit (subklinischem) VHF als Zufallsbefund durch weitere prospektive Studien zu therapeutischen Strategien untersucht werden [40, 41]. Eine Integration der Rhythmusdaten in ein umfassendes, patientenzentriertes und multidisziplinäres Therapiekonzept kann unter Einbeziehung von telemedizinischen Zentren künftig erfolgen. Somit könnten Daten über Vorhofflimmerlast als relevantes Diagnostikum für Therapieentscheidungen (z. B. Reablation) integriert und genutzt werden, zugleich aber auch in einem strukturierten und multimodalen Risikofaktormanagement therapiert und verbessert werden.

## Ausblick

Die aktuelle Studienlage zeigt ein verbessertes Verständnis der pathophysiologischen Hintergründe des VHF. Eine dynamischere Definition des VHF, im Sinne der Quantifizierung einer Vorhofflimmerlast, könnte das Erkrankungsstadium besser widerspiegeln. In der Evidenz finden sich jedoch noch deutliche Wissenslücken, insbesondere muss eine einheitliche Definition der Vorhofflimmerlast generiert und evaluiert sowie der zeitliche und pathophysiologische Zusammenhang zwischen erhöhter Vorhofflimmerlast und Schlaganfallrisiko durch weitere Studien untersucht werden. Der Einsatz von Wearables, insbesondere mit passiver Überwachungsfunktion, zur (semi-)kontinuierlichen Rhythmusaufzeichnung kann dazu beitragen, bestehende Wissenslücken zu schließen. Es muss jedoch frühzeitig der Einsatz notwendiger Versorgungsstrukturen, z. B. telemedizinischer Zentren, evaluiert werden.

## Fazit für die Praxis

- Eine genauere Charakterisierung von Vorhofflimmern, auch mithilfe der Quantifizierung der Vorhofflimmerlast, spielt sowohl auf wissenschaftlicher als auch auf klinischer Ebene eine immer wichtiger werdende Rolle.
- Wearables, insbesondere mit der Möglichkeit der passiven (semi-)kontinuierlichen Aufzeichnung können zur Quantifizierung der Vorhofflimmerlast herangezogen werden.
- PPG-basierte Aufzeichnungen mittels Wearables können vor allem zur Erfolgskontrolle einer antiarrhythmischen Therapie bei bereits etablierter Vorhofflimmerdiagnose herangezogen werden
- Vor einer breiten Anwendung von Wearables zur Charakterisierung der Vorhofflimmerlast sollte unter anderem die Frage nach notwendigen Versorgungsstrukturen zur Bearbeitung der Datenmenge geklärt werden.

## Abkürzungen

AHRE: Atriale Hochfrequenzepisoden
EKG: Elektrokardiogramm
OAK: Orale Antikoagulation
PPG: Photoplethysmographie
VHF: Vorhofflimmern
